# A mitochondrion-free eukaryote contains proteins capable of import into an exogenous mitochondrion-related organelle

**DOI:** 10.1098/rsob.220238

**Published:** 2023-01-11

**Authors:** Yi-Kai Fang, Zuzana Vaitová, Vladimir Hampl

**Affiliations:** ^1^ Charles University, Faculty of Science, Department of Parasitology, BIOCEV, Vestec 252 50, Czech Republic; ^2^ Institute of Organic Chemistry and Biochemistry of the Czech Academy of Sciences, Prague 160 00, Czech Republic

**Keywords:** mitochondrion-free eukaryote, hydrogenosome, protein import, evolution of protein targeting

## Abstract

The endobiotic flagellate *Monocercomonoides exilis* is the only known eukaryote to have lost mitochondria and all its associated proteins in its evolutionary past. This final stage of the mitochondrial evolutionary pathway may serve as a model to explain events at their very beginning such as the initiation of protein import. We have assessed the capability of proteins from this eukaryote to enter emerging mitochondria using a specifically designed *in vitro* assay. Hydrogenosomes (reduced mitochondria) of *Trichomonas vaginalis* were incubated with a soluble protein pool derived from a cytosolic fraction of *M. exilis*, and proteins entering hydrogenosomes were subsequently detected by mass spectrometry. The assay detected 19 specifically and reproducibly imported proteins, and in 14 cases the import was confirmed by the overexpression of their tagged version in *T. vaginalis*. In most cases, only a small portion of the signal reached the hydrogenosomes, suggesting specific but inefficient transport. Most of these proteins represent enzymes of carbon metabolism, and none exhibited clear signatures of proteins targeted to hydrogenosomes or mitochondria, which is consistent with their inefficient import. The observed phenomenon may resemble a primaeval type of protein import which might play a role in the establishment of the organelle and shaping of its proteome in the initial stages of endosymbiosis.

## Graphical abstract

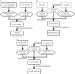

## 1. Introduction

One of the most crucial moments in the history of life occurred when the union between a eukaryotic ancestor and a prokaryotic ancestor of mitochondria was formed through endosymbiosis, eventually enriching the biosphere with a considerably more complex cellular type—a eukaryotic cell with a mitochondrion [1]. Mitochondria arose from an endosymbiont related to alpha-proteobacteria [2], and they are currently present in some form in almost every eukaryotic cell, where they perform many pivotal functions such as energy conservation [3], iron–sulfur cluster assembly [4], calcium homeostasis [5], reactive oxidation species metabolism [6] and others. Phylogenetic evidence indicates that proteins were lost, replaced and gained during the evolution of mitochondrial proteome while the organelle evolved and the host adapted to various environments [7]. Notable adaptations are represented by the mitochondrion-related organelles (MROs) present in eukaryotes living in low-oxygen environments [8,9]. Still, many pressing questions remain unanswered, including how the import of cytosolic proteins into the mitochondrion evolved [10]. More than 95% of mitochondrial proteins are synthesized as pre-proteins in the cytosol and then translocated into the organelles. This import depends on two important factors: protein complexes in mitochondrial membranes and sequence motifs present in the targeted proteins [11–14]. As extant mitochondria are well established in eukaryotic cells and cellular proteomes are adapted to this situation, the investigation into the origin of the protein targeting system is difficult using classical models.

*Monocercomonoides exilis* is a pear-shaped flagellate isolated from the intestine of *Chinchilla laniger* [15], and, like all its relatives from the group Metamonada, it thrives under low-oxygen pressure [16]. Unlike the other Metamonada species (e.g. *Trichomonas vaginalis* or *Giardia lamblia*), the genome of this protist encodes no hallmark mitochondrial proteins, such as components of mitochondrial import machinery or membrane carrier proteins, and so it is considered mitochondrion-free [17,18]. Even the mitochondrial type of the iron–sulfur cluster assembly system, which was considered one of the most conserved pathways among all MROs [4], was replaced by a prokaryotic alternative—the sulfur mobilization system [19]. The latter likely preadapted the lineage for the loss of mitochondria. We believe that this organism, which lost mitochondrion together with all accompanying proteins, represents a valuable system for the investigation of the early stages of protein import into the emerging protomitochondrial symbiont.

Questions about early mitochondrial evolution were previously addressed using artificial systems and prokaryotic models. Approximately one-quarter of randomly generated decapeptides starting with methionine were shown to deliver subunit IV of cytochrome oxidase into the yeast mitochondrion [20]. As much as 5% of *Escherichia coli* proteins are potentially predisposed for targeting to yeast mitochondria because they possess N-terminal extensions with similar properties to mitochondrion-targeting signals; however, this prediction was experimentally confirmed only for a single protein, YhaR [21]. Other studies showed that prokaryotic tail-anchored proteins insert into outer mitochondrial membranes [22] and that antimicrobial peptides can guide the reporter protein Venus to the mitochondrion or chloroplast in the green alga *Chlamydomonas reinhardtii* [23]. All these phenomena were considered as potential drivers of the early phase of the evolution of targeting into the mitochondrion because they facilitate initial binding to or crossing of the endosymbiont membrane, which had probably happened hand-in-hand with the gradual formation of the membrane translocases [24]. The aforementioned studies generally agree that some eukaryotic or prokaryotic proteins were predisposed to target a newly emerging organelle.

In this study, we have experimentally addressed the latter assumption on the model of the mitochondrion-free eukaryote *M. exilis*. Specifically, we probed whether the cytosolic extract of *M. exilis* contains proteins capable of binding and translocation into hydrogenosomes of *T. vaginalis*, mimicking the emerging mitochondria in our system. Hydrogenosomes are a category of MROs found in several anaerobic or microaerophilic protists, in which many mitochondrial functions, such as Krebs cycle and oxidative phosphorylation are absent [8], but a mitochondrial-type protein import mechanism [25,26] is retained in a simplified form. The close phylogenetic relationship between *T. vaginalis* and *M. exilis* and the relatively simple composition of hydrogenosomal protein translocons [26] makes it a potentially promising experimental system for this purpose.

## 2. Results

### 2.1. Assaying the ability of *Monocercomonoides exilis* proteins to enter hydrogenosomes *in vitro*

**Figure 1.**
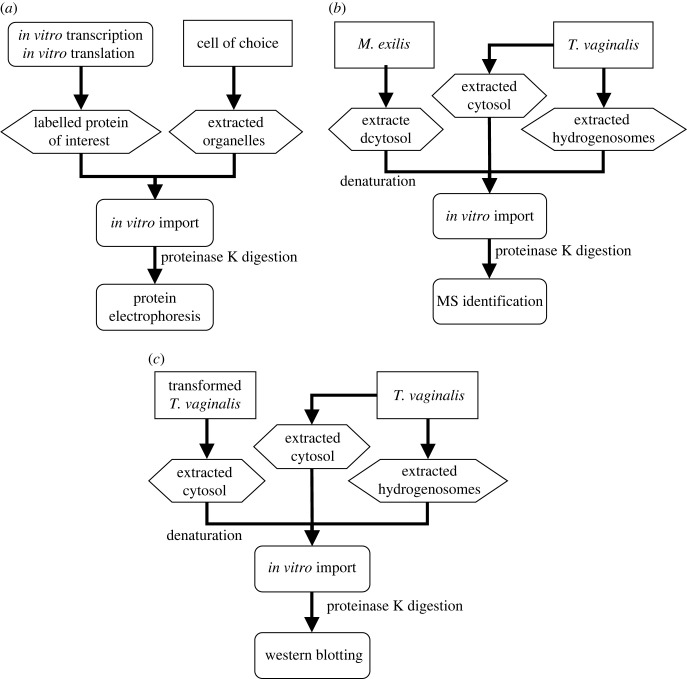
Scheme of experiment procedure. (*a*) Classical *in vitro* import is performed by incubation of *in vitro* synthesized protein with extracted target organelle, such as mitochondria or hydrogenosomes. (*b*) In our modified bulk version used for mining proteins that could be targeted into hydrogenosomes, the cytosolic proteins were extracted from *M. exilis*, denatured and incubated with hydrogenosomes supplemented with native cytosolic proteins extracted from *T. vaginalis*. After repurification and proteinase K treatment, hydrogenosomes were washed, and imported proteins were identified by MS. (*c*) To test the assay, we used *T. vaginalis* transformed with HA-tagged proteins as markers (TvFDP1 for cytosol and TvFTX for hydrogenosome). The cytosolic fraction of transformed *T. vaginalis* was extracted and incubated with hydrogenosomes extracted from wild-type cells. The HA signals in repurified hydrogenosomes treated with proteinase K were then detected by blotting.

*In vitro* import is a classic method to examine the ability of a protein to be imported into a specific organelle (figure 1*a*). We modified this method into a bulk variant that can be used for mining candidates for imported proteins from a complex mixture (figure 1*b*). In this setting, the tested proteins are not labelled but instead provided as a cytosolic extract. Because the import is performed into organelles isolated from a different organism (in our case, *T. vaginalis*), imported proteins can be detected in the repurified and proteinase K-treated organelles using mass spectroscopy (MS) and distinguished from the native organellar proteins. To increase the efficiency of the import, the proteins are linearized prior to import, and cytosolic chaperones are supplemented by the addition of the native *T. vaginalis* cytosolic fraction.

**Figure 2.**
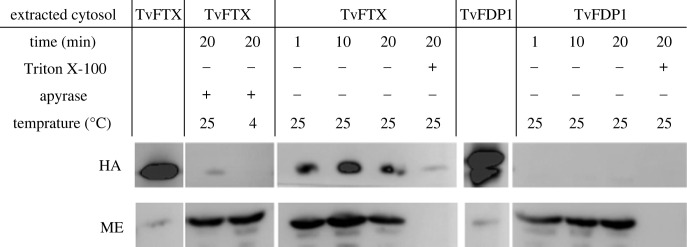
Validation of the import system. The import system was tested using the cytosol of *T. vaginalis* containing HA-tagged version of hydrogenosomal TvFTX or cytosolic TvFDP1 (figure 1*c*). *T. vaginalis* hydrogenosomes were incubated with denatured *T. vaginalis* cytosolic proteins for three time intervals (in minutes) at room temperature. Denatured cytosolic proteins without any treatment were also loaded to indicate the presence and location of HA-tagged proteins on the immunoblot. Proteinase K was added after incubation in all reactions to digest non-imported proteins. Triton X-100 was added in one 20 min incubation reaction to solubilize the membranes. The imported proteins were detected by anti-HA antibody and the hydrogenosomal protein, ME, was detected by antibody as an internal control. Apyrase was used in negative control to eliminate ATPs in the reaction.

To test this assay, we performed two parallel experiments using the cytosolic fraction of transformed *T. vaginalis* clones expressing haemagglutinin (HA)-tagged versions of either the hydrogenosomal protein frataxin (TvFTX) or the cytosolic flavodiiron protein 1 (TvFDP1) (figure 1*c*). The HA signal of TvFTX was observed in hydrogenosomes isolated from the wild-type after incubation, and it was resistant to proteinase K treatment (figure 2). At the same time, it was absent in the negative control incubated on ice, consistent with both the specific import of the nascent HA-tagged protein from the cytosol of the transformant and the absence of potential contamination by the hydrogenosomes from the transformant in the assay. In the parallel experiment with cytosolic protein TvFDP1, the HA signal remained in the cytosol as expected. These tests confirmed the functionality of the protocol and the robustness of the negative control on ice, which was used for all following experiments.

Three replicates of import assays using the *M. exilis* cytosolic fraction were performed, always in parallel with a positive control (HA-tagged TvFTX as above) and a negative control (no ATP supplement, incubated on ice). Repurified hydrogenosomes treated with proteinase K were used for MS protein identification. Altogether, 38 *M. exilis* proteins were identified in all three replicates together with 180 native *T. vaginalis* proteins (electronic supplementary material, table S1). Eight of the *M. exilis* proteins were detected also in negative controls (i.e. the import was non-specific) and 11 were not found in all replicates (i.e. the import was non-reproducible), which left 19 candidates for downstream validation (table 1).

**Table 1.** Potential hydrogenosome-targeting *M. exilis* proteins and their predicted and experimental localizations. CYT: cytosol; MIT: mitochondrion; NC: nucleus; PER: peroxisome; HYD: hydrogenosome; CYT/HYD: protein that was protected from proteinase K digestion and showed dual localization on western blotting.

ID	annotation	prediction								localization (cell fractions)
		CELLO	DeepLoc	iPSORT	MitoFates	MultiLoc2	PProwler	TargetP2	SVM	
MONOS_155	alcohol dehydrogenase (ADHE)	CYT	CYT	CYT	CYT	CYT	CYT	CYT	CYT	CYT/HYD
MONOS_1960	pyruvate phosphate dikinase 1 (PPDK1)	CYT	CYT	CYT	CYT	CYT	CYT	CYT	CYT	CYT
MONOS_3773	phosphoglycerate kinase (PGK)	CYT	CYT	CYT	CYT	CYT	CYT	CYT	HYD	CYT/HYD
MONOS_4492	enolase (ENO)	CYT	CYT	CYT	CYT	CYT	CYT	CYT	CYT	CYT/HYD
MONOS_4759	tubulin alpha chain (TUB alpha)	CYT	CYT	CYT	CYT	CYT	CYT	CYT	CYT	CYT/HYD
MONOS_5021	glyceraldehyde-3-phosphate dehydrogenase (GAPDH1)	CYT	CYT	MIT	CYT	CYT	MIT	CYT	HYD	CYT/HYD
MONOS_5294	glyceraldehyde-3-phosphate dehydrogenase (GAPDH2)	CYT	CYT	MIT	CYT	CYT	MIT	CYT	CYT	CYT/HYD
MONOS_6112	conventional actin (ACT)	CYT	CYT	CYT	CYT	CYT	CYT	CYT	CYT	CYT/HYD
MONOS_6571	heat shock protein 90 (HSP90)	CYT	CYT	CYT	CYT	CYT	CYT	CYT	CYT	CYT/HYD
MONOS_6709	pyruvate-ferredoxin oxidoreductase (PFO2)	CYT	CYT	CYT	CYT	CYT	CYT	CYT	CYT	CYT
MONOS_7522	phosphoglycerate dehydrogenase (PGDH)	CYT	CYT	CYT	CYT	MIT	CYT	CYT	HYD	CYT/HYD
MONOS_9400	tubulin beta chain (TUB beta)	CYT	CYT	CYT	CYT	CYT	CYT	CYT	CYT	CYT/HYD
MONOS_9530	pyruvate-ferredoxin oxidoreductase (PFO3)	CYT	CYT	CYT	CYT	CYT	CYT	CYT	CYT	CYT
MONOS_9694	coronin (CRN)	CYT	CYT	CYT	CYT	CYT	CYT	CYT	CYT	CYT/HYD
MONOS_9745	cyclophilin (CYP)	CYT	CYT	CYT	CYT	CYT	CYT	CYT	HYD	CYT/HYD
MONOS_10289	acetaldehyde-CoA/alcohol dehydrogenase (ADHE)	CYT	CYT	CYT	CYT	CYT	CYT	CYT	CYT	CYT
MONOS_10363	acetyl-CoA synthetase (ACS)	CYT	PER	CYT	CYT	CYT	CYT	CYT	HYD	CYT/HYD
MONOS_11342	Adenosine deaminase (ADA)	CYT	CYT	CYT	CYT	CYT	CYT	CYT	CYT	CYT
MONOS_13094	hypothetical protein (HYP)	CYT	CYT	CYT	CYT	NC	CYT	CYT	CYT	CYT/HYD

### 2.2. Validation of protein import by expression in *Trichomonas vaginalis*

**Figure 3.**
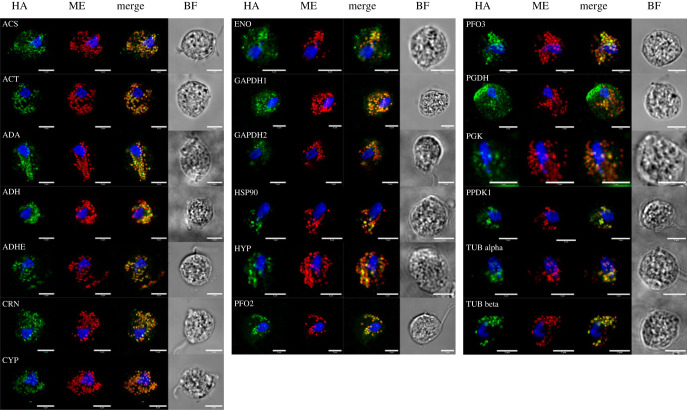
Localization of selected *M. exilis* proteins in *T. vaginalis* by immunofluorescence. Selected *M. exilis* proteins with a C-terminal HA tag were expressed in *T. vaginalis* under a weak promotor. The immunofluorescence microscope images show hydrogenosomes labelled by anti-ME antibody in red and the localization of HA-tagged proteins in green. Scale bars are 5 µm. BF: bright field. ACS: acetyl-CoA synthetase (ADP-forming); ACT: actin; ADA: adenosine deaminase; ADH: alcohol dehydrogenase; ADHE: bifunctional acetaldehyde-CoA/alcohol dehydrogenase; CRN: coronin; CYP: cyclophilin; ENO: enolase; GAPDH: glyceraldehyde-3-phosphate dehydrogenase; HSP90: heat shock protein 90; HYP: hypothetical protein; PFO: pyruvate-ferredoxin oxidoreductase; PGDH: phosphoglycerate dehydrogenase; PGK: phosphoglycerate kinase; PPDK1: pyruvate phosphate dikinase 1, TUB: tubulin.

To validate the targetability of the 19 candidate proteins into hydrogenosomes, their C-terminally HA-tagged versions were transiently expressed in *T. vaginalis* under a weak ferredoxin promoter to minimize overexpression artefacts. Immunofluorescence on methanol-fixed cells, which has the advantages of intensive staining and low cytoplasmic backgrounds [27,28], was used to localize the proteins in the cell. All selected candidates showed a pattern of at least partial co-localization with the hydrogenosomal matrix protein malic enzyme (ME), suggesting that these proteins may indeed localize in or at the surface of hydrogenosomes (figure 3).

**Figure 4.**
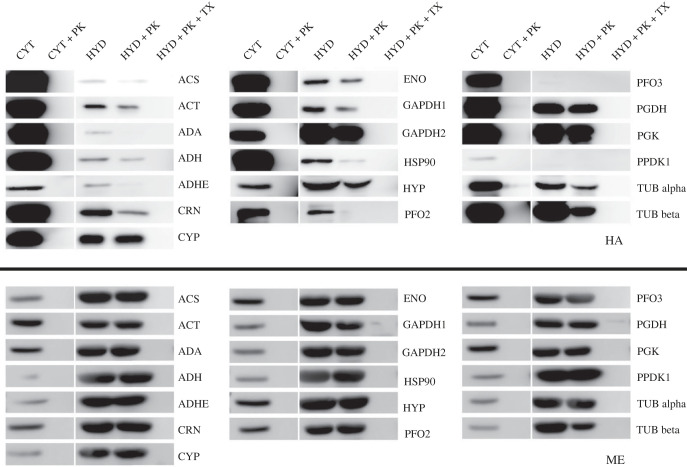
Localization of selected *M. exilis* proteins in *T. vaginalis* by western blot on cell fractions. The cytosolic (CYT) and hydrogenosomal (HYD) fractions were extracted from HA-tagged protein-transformed *T. vaginalis*. The same amount of extracted hydrogenosomal proteins was treated with proteinase K (PK) to remove surface-associated proteins. Triton X-100 (TX) was used to solubilize membranes to reveal that HA signals are not resistant to PK digestion. After development with anti-HA antibody (upper panel), the same nitrocellulose membrane was then stripped and re-hybridized with anti-ME antibody (lower panel).

To further verify the conclusion from immunofluorescence, and to confirm that the proteins were indeed internalized into the organelles and not surface-associated, we compared the HA signals from the cytosolic and hydrogenosomal fractions of transformed *T. vaginalis* using western blotting (figure 4). The internalization of the protein was assessed by proteinase K protection assays with and without detergent. For each fraction, the same amount of extracted proteins was treated with the same dosage of proteinase K. In most cases, the majority of HA signal remained in the cytosolic fraction. For 17 of 19 proteins, the HA signal was detectable in the hydrogenosomal fractions, while for pyruvate phosphate dikinase 1 (PPDK1) and pyruvate-ferredoxin oxidoreductase (PFO3), it was not. The absence of PPDK1 in the hydrogenosome fraction is probably caused by the low expression of its HA-tagged version in the fractionated *T. vaginalis* cells. For PFO3, the reason for the absence of a signal in the hydrogenosomal fraction is unclear. Both proteins were removed from the list of imported proteins. For 14 of the 17 proteins, a greater or lesser part of the signal was protected from proteinase. Similar patterns were observed for the ME control, only in this case always the majority of the signal was protected. Three proteins, adenosine deaminase (ADA), acetaldehyde-alcohol dehydrogenase (ADHE) and PFO2 were not protected from proteinase K digestion, suggesting they were not entering hydrogenosome but associated with the hydrogenosome surface. These three proteins were also removed from the list of imported proteins.

### 2.3. Features of the hydrogenosome-targeted *Monocercomonoides exilis* proteins

Most of the imported proteins have well-annotated functions, which encompass glycolytic and other enzymes (e.g. PFO, ADHE), regulatory proteins (cyclophilin, CYP) and parts of the cytoskeleton (tubulin, actin and coronin) (table 1). This functional diversity is not unexpected, as it is the proteins' structural properties rather than their functions that determine their targeting. In order to assess the structural properties potentially relevant to protein import, we assessed the ability of mitochondrial targeting for all candidates using multiple predictors, such as MitoFates, TargetP2, MultiLoc2, iPSORT and PProwler. Only two glyceraldehyde-3-phosphate dehydrogenases (GAPDHs) were predicted to have potentially mitochondrial localization by iPSORT and PProwler, and only phosphoglycerate dehydrogenase (PGDH) by MultiLoc2 (table 1). The remaining proteins were predicted to be mostly cytosolic, one peroxisomal and one nuclear.

Prediction of targeting into MROs, including *T. vaginalis* hydrogenosomes, is known to be difficult, as the N-terminal signals are non-canonical, and internal signals are involved as well [29–32]. A more sensitive machine learning method was used to distinguish hydrogenosomal from cytosolic proteins in *T. vaginalis*, with a specificity based on experimental validation around 60% [33]. We built on this previous study and predicted subcellular localizations of *M. exilis* candidates using a support vector machine (SVM) classifier trained on *T. vaginalis* hydrogenosomal and cytosolic proteins. We used the same protein features as in Burstein *et al.* [33], but did not include genetic and phylogenetic features. The accuracy of the trained classifier for *T. vaginalis* was still not optimal as it correctly identified only 40/42 (95.24%) cytosolic and 12/20 (60%) hydrogenosomal proteins in the testing phase. The same classifier classified only five (acetyl-CoA synthetase (ACS), CYP, GAPDH1, phosphoglycerate kinase (PGK) and PGDH) of the 14 imported *M. exilis* proteins (35.71%, column SVM of table 1) as hydrogenosomal. With the exception of ACS, SVM-predicted hydrogenosome-targeted proteins were imported into hydrogenosomes with relatively good efficiency in our assay (figure 4).

In summary, few of the imported *M. exilis* proteins were recognized as hydrogenosomal, either by available predictors or by an SVM classifier trained on *T. vaginalis* hydrogenosomal proteins. This suggests that these proteins do not exhibit features considered by these tools as important for targeting and it is consistent with a low-efficiency import and the absence of transit peptide processing observed in western blots (figure 4).

## 3. Discussion

The mitochondrial endosymbiosis was the major transition in life's evolutionary history from which stem all extant eukaryotic cells. The majority of mitochondrial proteins in the cell are now synthesized in the cytosol and posttranslationally translocated into the organelle by a complex mechanism involving dozens of proteins [11]. This machinery was apparently already functional in the last eukaryotic common ancestor, and its establishment likely evolved hand-in-hand with the features of the targeted proteins responsible for their recognition and import [24]. Although the organelle-targetability of most proteins probably evolved and was optimized by natural selection after the endosymbiosis, it cannot be excluded that, in some fraction of proteins, this capability was already present as a product of neutral evolution. Such proteins could readily contribute to the emerging organellar proteome and affect both the biochemistry and formation of the protein import mechanism of this new compartment. Here we investigate this crucial evolutionary moment by composing an artificial system *in vitro* consisting of the cytosol of the mitochondrion-free eukaryotic cell and a heterologous MRO. In this system, the cytosolic proteome of *M. exilis* represents the cytosol of the hypothetical amitochondriate cell prior to endosymbiosis. The potential caveat is the secondary amitochondriate status of *M. exilis*, due to which it cannot be strictly excluded that features of some proteins are still affected by the past presence of the mitochondrion. This is, however, very unlikely, as the loss of mitochondria in the oxymonad lineage happened at least a 100 Ma, and more importantly, there is no evidence for the presence of any originally mitochondrial protein in the current proteome of *M. exilis* (Novak *et al.* in prep [34]). The hydrogenosome of *T. vaginalis* is obviously remote from the original mitochondrial endosymbiont, but its translocation system is arguably simpler than the one in aerobic mitochondria [26], so it may mimic the primitive situation well. Therefore, we believe the composed system is suitable to test at least some hypotheses, such as the origin of protein features facilitating the targeting by neutral evolution.

Using our artificial import system, we found and validated 14 proteins capable of import into hydrogenosomes, which represents less than 1 ‰ of the total proteome consisting of 18 152 predicted proteins [18]. No indication of N-terminal cleavage after the import was observed in any of them, and in most cases, the majority of the expressed proteins remained in the cytosol. This suggests that, although specific, the import of these proteins is usually inefficient, which is not unexpected for neutrally evolved delivery and consistent with the hypothesis proposing that protein import was initiated by constitutive minor mistargeting [10]. Interestingly, the set of imported proteins does not seem to be random. It is clearly enriched with enzymes for glycolysis (enolase, GAPDH), extended glycolysis (ACS), metabolism connected to glycolysis (PGDH) and fermentation (ADHE). Most of these proteins are abundant and the higher concentration of protein outside the organelle could help the inefficient import system to deliver a detectable amount of proteins inside. This phenomenon may provide adaptive value, as some of the abundant cellular enzymes may quickly become involved in the organelle's metabolism, and their import efficiency may subsequently be optimized by natural selection.

Naturally, we wondered if these proteins contain any common feature that helped them to enter hydrogenosomes. Clearly, none of them possesses typical hydrogenosomal N-terminal targeting sequences (NTS) [35], and only three proteins were predicted to have mitochondrial localization. The NTS was previously considered essential for the targeting of hydrogenosomal proteins; however, examples of thioredoxin reductase and phosphofructokinase showed that proteins lacking an NTS can also be imported, probably due to an unknown internal signal [29–32], and this NTS-independent import was proposed to be a relic from the early phases of endosymbiosis [32]. Machine learning provides a solution for how to adjust predictions to a specific organism, and it has been already used for predicting localization in *T. vaginalis* hydrogenosomes, although the accuracy was not optimal [33]. We retrained the classifier of Burstein *et al.* [33] on an updated set of *T. vaginalis* hydrogenosomal proteins identified in our measurements, but reached only a 60% accuracy in predicting hydrogenosomal localization in the test phase. With the same setting, the predictor classified five of the 14 proteins of *M. exilis* as hydrogenosomal. Altogether this shows that none of the predictors or classifiers was able to accurately identify *M. exilis* proteins that were shown to be imported experimentally. Most likely, features considered by the software to be important for targeting are not present, and the import is driven by other characteristics hidden from us, higher abundance of some of these proteins in the cytosol or a combination of the two.

In conclusion, we observe that a small fraction of proteins in a mitochondrion-free cell can associate with and enter an organelle, the hydrogenosome of *T. vaginalis*, with which they have never previously been in contact. Although this ‘accidental’ import is in most cases inefficient, it seems both specific and reproducible. It is tempting to speculate that similar phenomena may have contributed to the formation of the proteomes of mitochondria and plastids in the early stages of endosymbiosis and initiated the establishment of each as organelles. In the course of evolution, the efficiency and specificity of import would then have gradually improved via natural selection by coevolution of the protein import complexes on the membranes and of the structural characteristics of the imported proteins.

## 4. Materials and methods

### 4.1. *Monocercomonoides exilis* cytosol extraction

*Monocercomonoides exilis* PA203 was maintained in modified TYSGM medium at 37°C as previously described [17]. The collected cells were then washed with culture medium (without serum addition) and suspended in a protease-inhibitor-containing import buffer (250 mM sucrose, 10 mM Tris, 2 mM potassium phosphate pH 7.4, 25 mM KCl, 10 mM MgCl_2_, 0.5 mM EDTA, 1 mM DTT, 3% bovine serum albumin). The suspension was then sonicated in 1 s pulses at 20% amplitude, alternating with 1 s pauses, until greater than 90% cells were broken. Cell lysate was ultra-centrifuged (120 000 x g for 20 min at 4°C) and the supernatant representing the cytosolic fraction was collected. The purified cytosolic fraction was then precipitated by methanol and chloroform and resolved in urea buffer (7 M urea, 30 mM MOPS-KOH pH7.2, 1 mM DTT). The protein concentration was determined by a bicinchoninic acid (BCA) assay.

### 4.2. Purification of *Trichomonas vaginalis* hydrogenosomes and cytosol

The hydrogenosomes of *T. vaginalis* were isolated as previously described [36]. Briefly, cells in mid-logarithmic phase were collected and sonicated to disrupt the cells. The large-granule fraction (LGF) was separated from the crude cytosolic fraction by centrifugation and suspended in 45% Percoll solution for ultracentrifugation. The extracted hydrogenosomes were washed twice and resuspended in import buffer. The supernatant above the LGF fraction was used to prepare the cytosolic fraction by ultracentrifugation (120 000 x g for 20 min at 4°C). The concentration of collected proteins was determined by a BCA assay.

### 4.3. *In vitro* protein import assay

To test the assay, 5 µg of denatured cytosol from TvFDP1 or TvFTX transformed *T. vaginalis* cells were incubated with 20 µl of purified wild-type hydrogenosomes (200 µg) and 10 µl of 0.2 M ATP at room temperature for 1, 10 or 20 min. For the negative control, apyrase (20 U ml^−1^) was used to eliminate ATP as previously described [32], and incubation with hydrogenosomes was carried out on ice without supplemented ATP. After incubations, non-imported proteins were digested by 10 µg of proteinase K on ice for 15 min, and digestion was then stopped by the use of 1 mM final concentration phenylmethylsulfonyl fluoride for 15 min on ice. Hydrogenosomes were repurified by centrifugation (20 000 x g for 10 min at 4°C) and washed twice before suspended in import buffer. Three replicates of the main import assay with *M. exilis* cytosol were performed under the same conditions as above, except that only the purified hydrogenosomes were incubated with 5 µg of denatured *M. exilis* cytosol supplemented with 5 µg of purified native *T. vaginalis* (wild-type) cytosol. The hydrogenosomes were repurified and used for MS identification. In the case of the Triton X-100 treatment, 0.5% final concentration of Triton X-100 was added into the reaction during the proteinase K treatment; the proteins were then precipitated by methanol and chloroform and resolved by SDS-PAGE sample buffer for electrophoresis.

### 4.4. Mass spectrometry sample preparation and data acquisition

Protein samples for MS were prepared as previously described [37]. Briefly, samples were precipitated and dissolved in triethylammonium bicarbonate with 1% sodium deoxycholate. Proteins were then digested by trypsin, acidified with trifluoroacetic acid and desalted using in-house made stage tips packed with C18 discs [38]. Nano reversed-phase columns were used for LC/MS analysis. Tandem MS was performed by isolation at 1.5 Th with the quadrupole, HCD fragmentation with normalized collision energy of 30 and rapid scan MS analysis in the ion trap. The MS2 ion count target was set to 104 and the maximum injection time was 35 ms. Only those precursors with charge states 2–6 were sampled for MS2. The dynamic exclusion duration was set to 45 s with a 10 ppm tolerance around the selected precursor and its isotopes.

### 4.5. Analysis of mass spectrometry data

All data were analysed and quantified with MaxQuant software (version 1.6.10.43, RRID: SCR_014485) as previously described [37]. The Andromeda search engine was used for the MS/MS spectrum search against the *M. exilis* database (acquired from GiardiaDB, https://giardiadb.org/giardiadb/app) and *T. vaginalis* database (from TrichDB, https://trichdb.org/trichdb/app). Quantifications were performed with the label-free algorithm in MaxQuant [39]. Data analysis was performed using Perseus 1.6.13.0 software (RRID: SCR_015753) [40]. The MS proteomics data have been deposited to the ProteomeXchange Consortium via the PRIDE partner repository (RRID: SCR_004055) [41] with the dataset identifier PXD035701 and 10.6019/PXD035701.

### 4.6. *Trichomonas vaginalis* transformation, immunofluorescence microscopy and western blot

Selected proteins were amplified by PCR from *M. exilis* cDNA and cloned into plasmid pTagVag2 with the *T. vaginalis* ferredoxin (TVAG_003900) promoter and a C-terminal HA tag. The transformation of *T. vaginalis* was performed and selected with 200 mg ml^−1^ of G418 as described previously [42]. The transformed *T. vaginalis* cells were collected and then fixed by methanol at −20°C for 10 min. The HA-tagged proteins were detected by a monoclonal rat antibody (Roche, cat. no.: 11867423001, RRID: AB_390918), and the hydrogenosomal protein, ME, was detected by a polyclonal rabbit antibody. Secondary Alexa Flour antibodies (Thermo Fisher Scientific, cat. no. A-21207, RRID: AB_141637; cat. no. A-21470, RRID: AB_2535873) were used for visualization of the target proteins. The acquired images were deconvoluted using Huygens and then processed by ImageJ. Western blots were performed on cytosolic and hydrogenosomal fractions isolates, proteinase K and Triton X-100 treated as described above.

### 4.7. *In silico* analyses of protein features

An SVM was applied for protein classification [43]. The classifier was trained and tested on a dataset of 169 cytosolic and 80 hydrogenosomal proteins from *T. vaginalis*, the subcellular localization of which were confirmed experimentally in our and/or previous experiments [36], and is reported in UniProtKB (RRID: SCR_004426) (electronic supplementary material, table S2). One hundred and eighty-seven (75%) proteins randomly selected from the set were used for training, and the remaining 62 for testing of the trained classifier. The features (such as charge and polarity) and amino acid composition of the proteins were selected as in Burstein *et al.* [33].

## Data Availability

The data are provided in the electronic supplementary material [44].
